# A case of iatrogenic intussusception in adults: a rare case report

**DOI:** 10.1186/s12893-021-01268-2

**Published:** 2021-05-29

**Authors:** Qiang Hu, Jianfeng Shi, Yuanshui Sun, Jinfeng Shi

**Affiliations:** Department of General Surgery, Tongde Hospital of Zhejiang Province, 234 Gucui RD, Hangzhou, 310012 China

**Keywords:** Intussusception, Long intestinal tube, Intestinal obstruction, Colon tumor, Case report

## Abstract

**Background:**

Intussusception has a low incidence rate in adults. Many cases in adults are caused by tumors. Intussusception results from conditions other than tumors are uncommon. This is the first case report about intussusception that occurred after removing a long intestinal tube (LT).

**Case presentation:**

A 69-year-old female complained of “recurrent abdominal pain with reduced flatus passage and frequency of bowel movement for 10 days” was admitted to the hospital. Plain abdominal radiography and abdominal CT upon admission showed intestinal obstruction. The patient’s abdominal pain was not relieved after symptomatic treatments, which involved fluid and electrolyte replacement, LT placement, spasmolytic agents, and analgesics. Hence, surgical exploration was carried out. The patient had a good recovery postoperatively. No abdominal pain or bloating developed after food intake. The patient passed flatus and had bowel movements later. On postoperative day 9, the LT was removed. On the 10th day, the patient suddenly developed abdominal distension and acute abdominal pain. Emergency abdominal CT showed small bowel intussusception. Surgical exploration was then performed. Severe small bowel dilatation located at 1.5 m from the ligament of Treitz was found during the procedure. Intussusception at the site was observed. No color change of the intestinal wall was detected, suggesting that no necrosis was present. So, a manual reduction was done. The patient was discharged on postoperative day 6.

**Conclusions:**

This case serves as a warning that the simple action of pulling out the LT might also cause serious complications, which should be given more attention.

## Background

Unlike intussusception in children, the incidence of intussusception in adults is extremely low, and most of them are secondary [1]. In previous reports, adult intussusception occurred is mostly secondary to intestinal tumors, and other causes leading to adult intussusception are rarely reported in clinic settings. This paper reviewed and summarized the diagnosis and treatments of one case of adult intussusception caused by an LT removal in our hospital. Relevant issues were discussed and analyzed.

## Case presentation

**Fig. 1:**
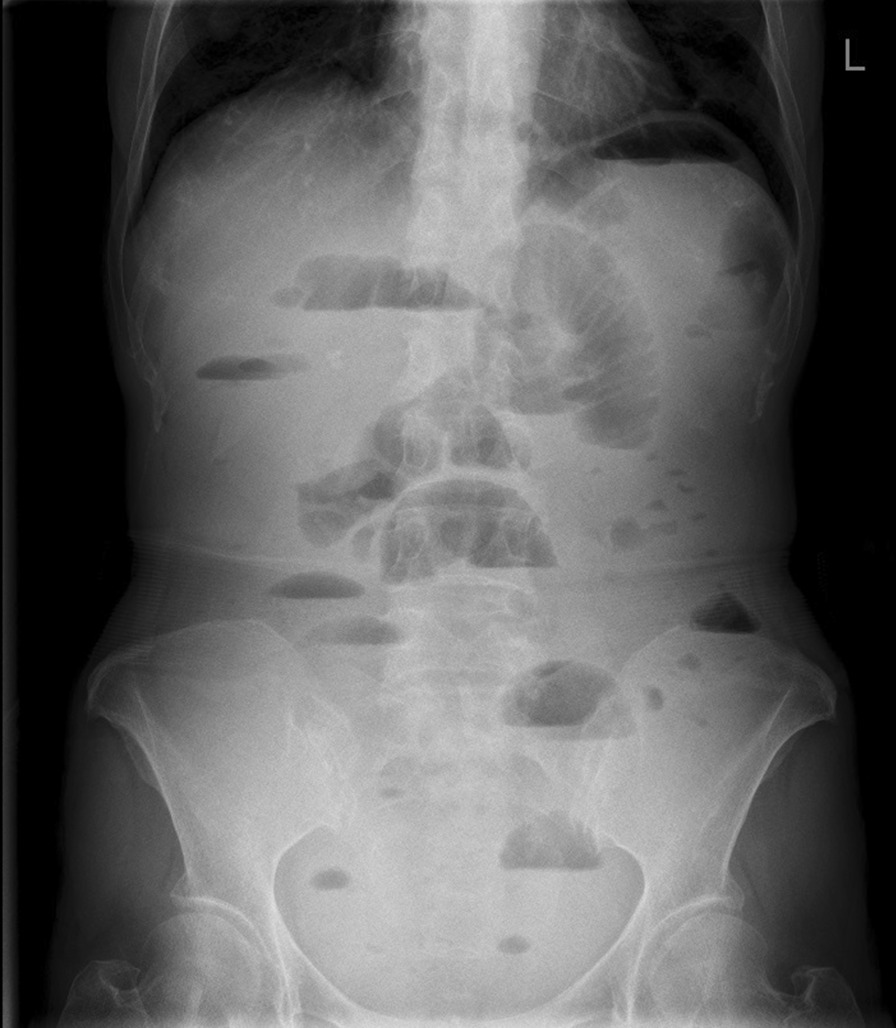
Plain abdominal radiography suggest intestinal obstruction

**Fig. 2:**
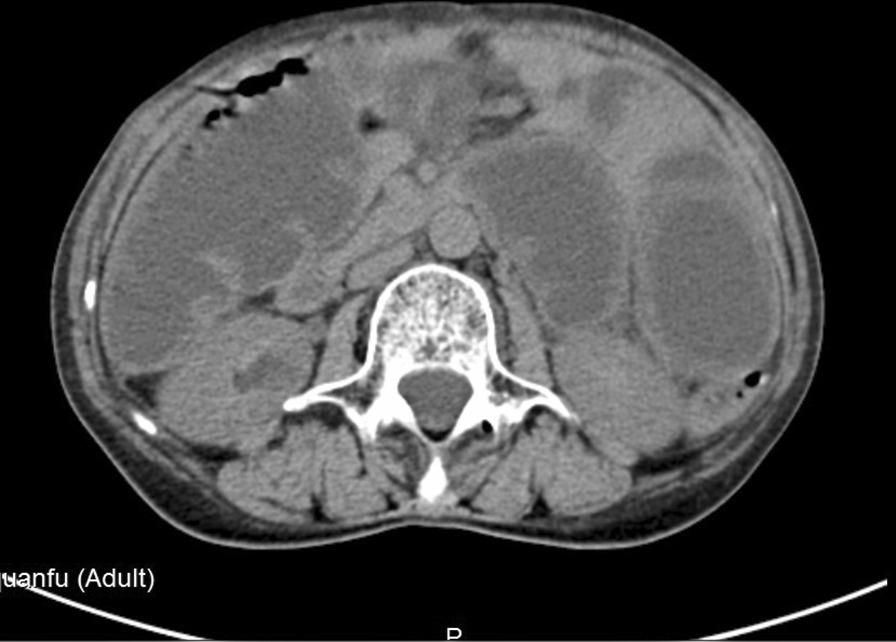
CT of the abdomen reveals incomplete colonic obstruction

**Fig. 3:**
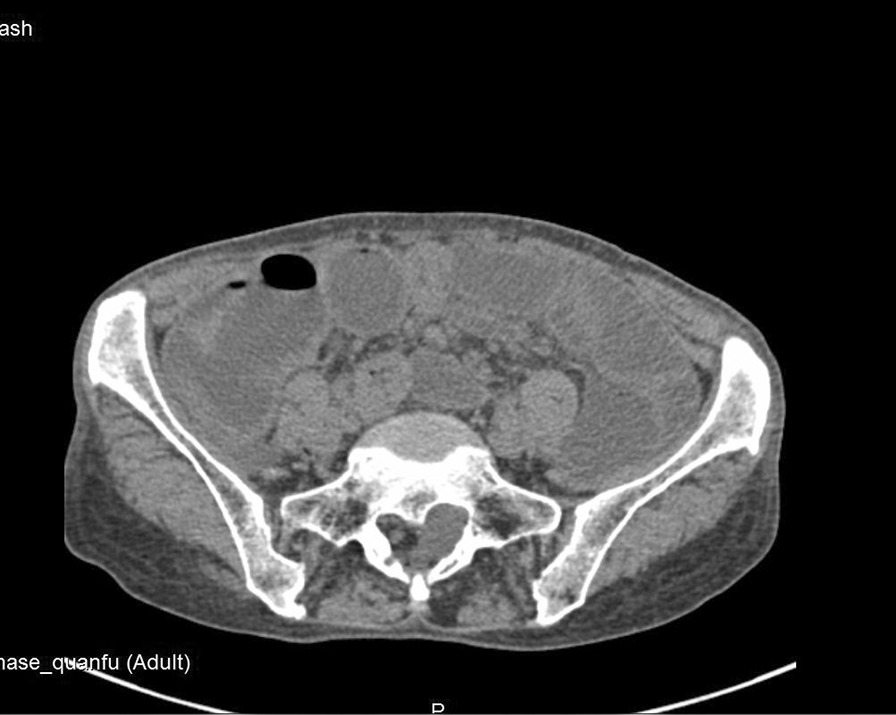
No intussusception was found on abdominal CT before operation

A 69-year-old female patient complained of “recurrent abdominal pain with reduced flatus passage and frequency of bowel movement for 10 days” was admitted to the hospital. Physical examination: slight abdominal distension, hyperactive bowel sounds (about 6 times/min), no tenderness or rebound pain, shifting dullness, no high-pitched bowel sounds, or gurgling. The liver and spleen under the rib cage were not palpated. Laboratory results: WBC: 5.9 × 10E9/L, Neutrophile (%): 66.9, Hemoglobin: 134 g/L, C-reactive protein (CRP): 10.53 mg/L, Carcinoembryonic antigen (CEA): 19.30ng/ml, CA 125: 92.90 U/ml, CA 72: 96.64 IU/ml. Radiological findings: Plain abdominal radiography: obvious pneumatosis in the abdominal cavity with intestinal dilatation (Fig. 1), Abdominal CT: incomplete large bowel obstruction (the obstruction point might locate at the splenic flexure ) (Fig. 2). No intussusception was found (Fig. 3). Past medical history: On April 2, 2018, laparoscopic radical gastrectomy was performed in our hospital for cardia adenocarcinoma. The postoperative recovery was satisfactory. TNM stage was T4N3M0 postoperatively. The patient received six times of SOX (180 mg oxaliplatin + 50 mg S-1) regimen on April 23, 2018, May 16, 2018, June 5, 2018, July 3, 2018, August 1 2018, and September 6, 2018. She received S-1 (50 mg) monotherapy on September 25, 2018, November 13, 2018, January 8, and March 26, 2019. Further courses of chemotherapy were terminated because of patient refusal.

**Fig. 4:**
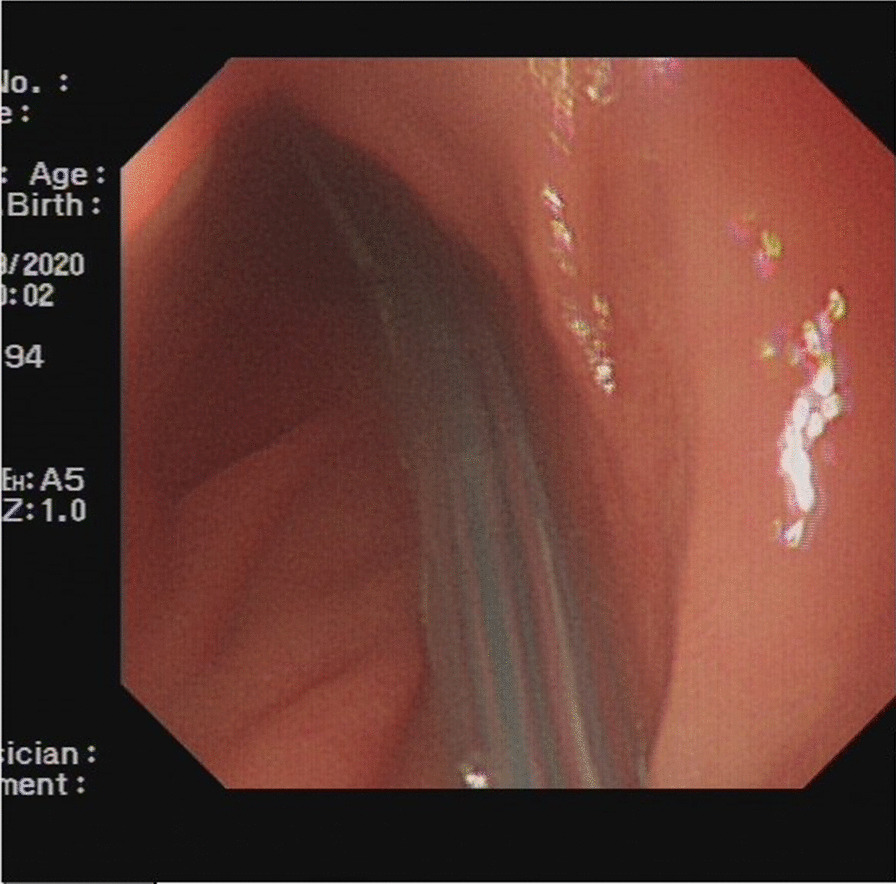
Insertion of intestinal obstruction catheter

**Fig. 5:**
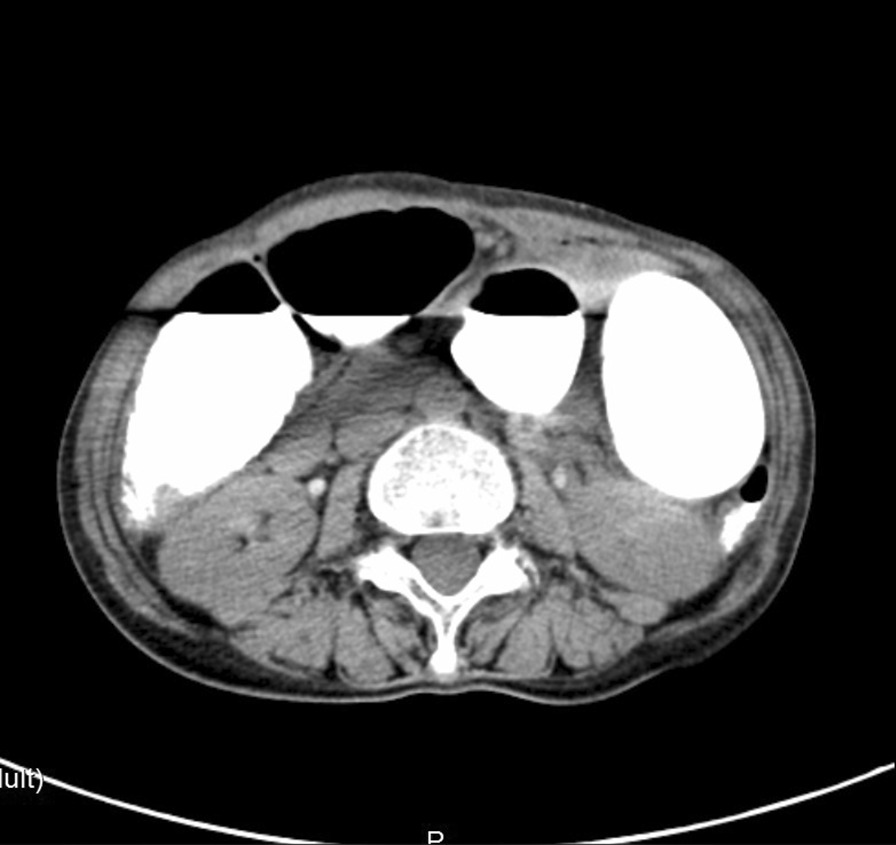
Reexamination of abdominal CT showed that the intestinal obstruction was not significantly relieved

Upon admission, the patient received symptomatic treatments, which consisted of fluid and electrolyte replacement, LT placement, spasmolytic agents, and analgesics. The LT (16DBR 3000T0,16Fr, Dalian Create Medical Products Co., Ltd.) was placed on September 30, 2020 (Fig. 4). Abdominal CT after the procedure showed no significant relief of the bowel obstruction (Fig. 5). Surgical exploration was performed on October 12, 2020, then. During the operation, the colon proximate to the splenic flexure was dilated. The wall of the left part of the transverse colon was thickening due to chronic oedema. Part of the wall was invaded by the tumor. The distal descending colon and sigmoid colon were empty and collapsed. No intussusception was found during the exploration. After resection of the lesion by removing the part of the descending colon proximate to the site of invasion and the entire transverse colon, the remainder of descending colon was brought close to the ascending colon, and side-to-side anastomosis was done using the stapler. The bowel openings were evenly aligned and closed using the linear cutter and occluder.

**Fig. 6:**
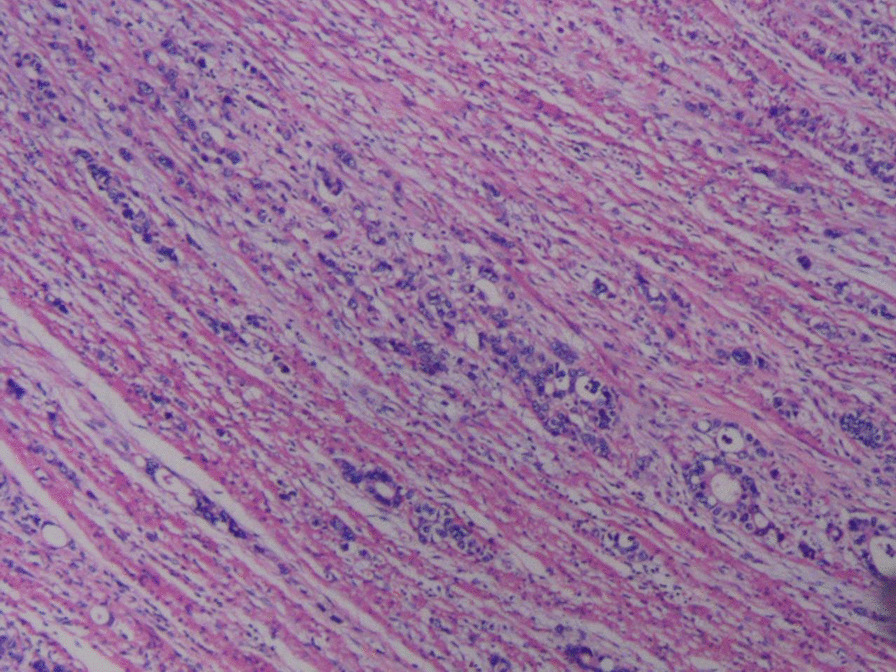
Pathology after colonic surgery suggests moderate to poorly differentiated adenocarcinoma (HE*100)

**Fig. 7:**
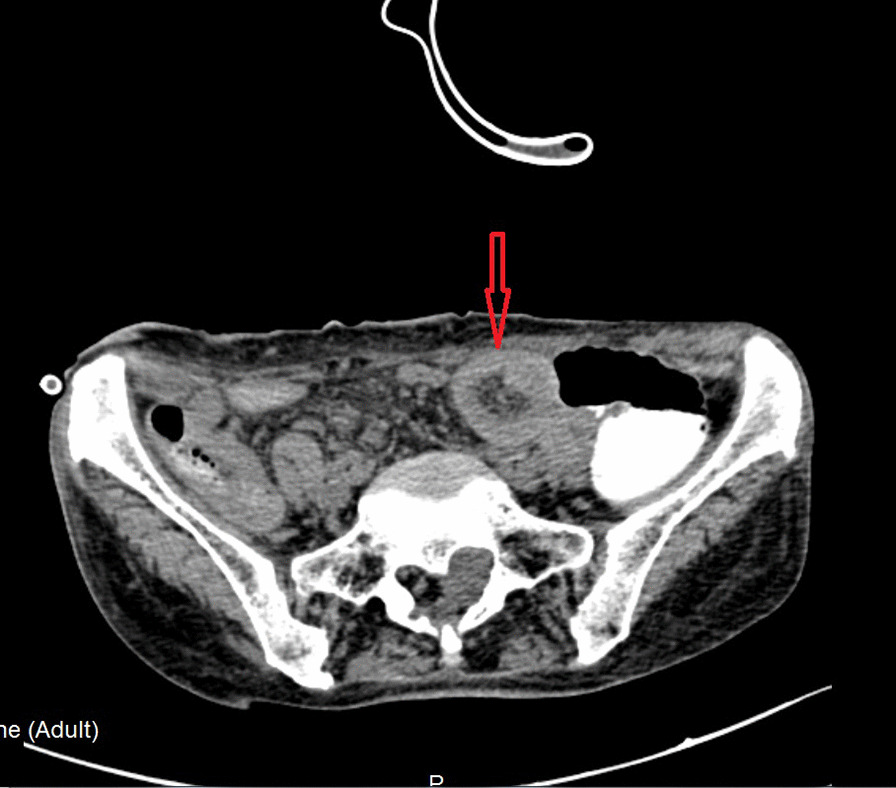
CT of the abdomen suggests intussusception(concentric circle sign)

**Fig. 8:**
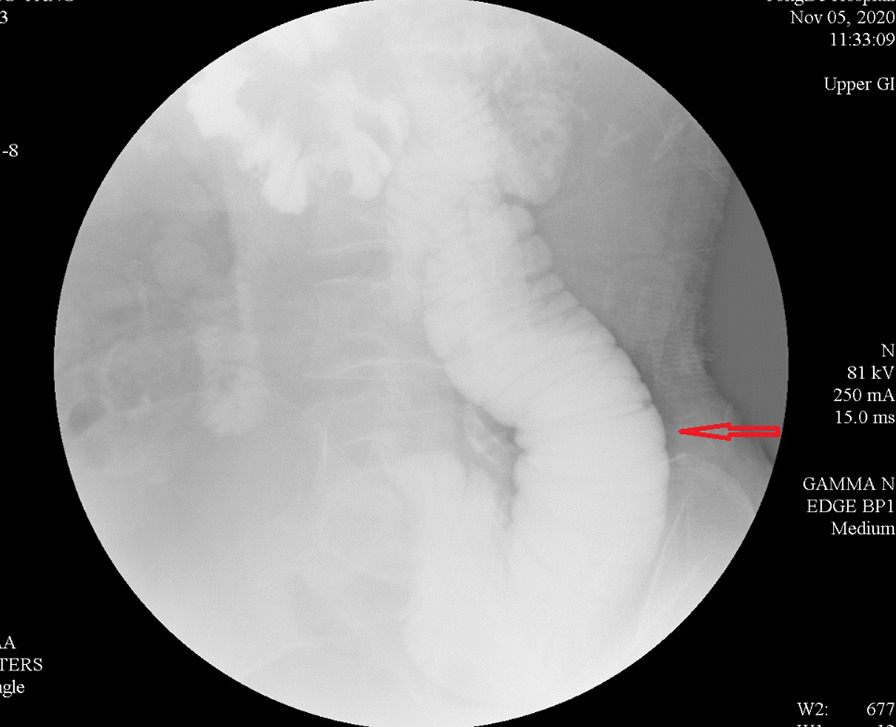
Gastrointestinal radiography suggests small bowel obstruction

**Fig. 9:**
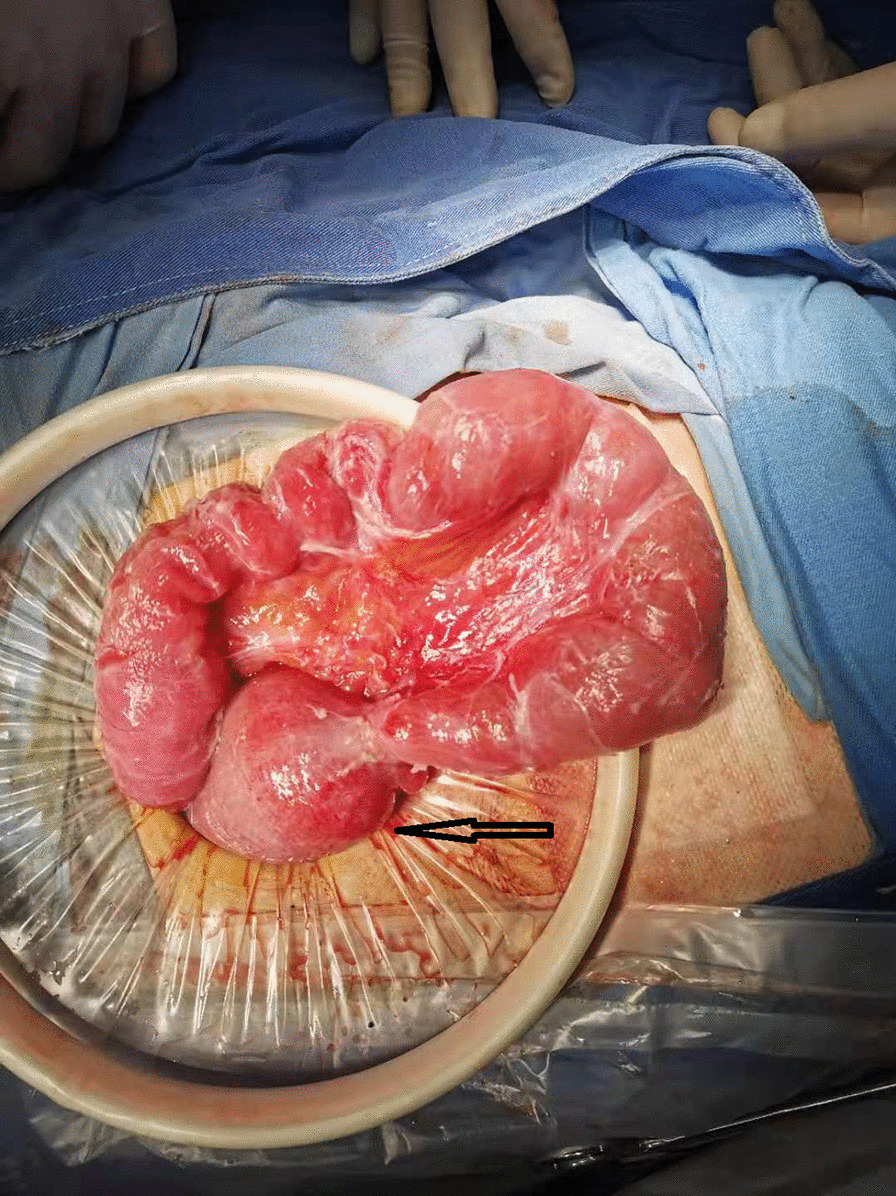
Site of intussusception during operation

There are two main reasons why we did not pull out the LT before the surgical intervention: (1) the LT helps with gastrointestinal decompression, which prevents postoperative intestinal dilatation due to poor intestinal function (2) the tube helps with intestinal splinting, which reduces the recurrence of bowel obstruction postoperatively. Postoperative pathology: moderately to poorly differentiated adenocarcinoma. Considering the patient’s past medical history and immunohistochemistry results, the patient’s condition met the diagnostic criteria of colonic metastasis of cardia adenocarcinoma (Fig. 6). Genetic testing: Desmin(+), MSH2(+), MSH6(+), MLH1(+), PMS2(+), Muc-2(+), Muc-6(−), Muc-5AC(+), CD34(+), S-100(+), P53(+), Ki-67(+,20 %), CerbB-2(−), CK7(+), CK20(+), CDX-2(+). After the operation, the patient had nothing by mouth and received parenteral nutrition. On postoperative day 2, the patient passed flatus and was suggested to start oral water intake. On postoperative day 4, the patient had bowel movements. On postoperative day 7, the patient was placed on the full liquid diet. No abdominal pain and distension developed after food intake. On postoperative day 9, the LT was removed. On postoperative day 10, the patient suddenly developed abdominal distension and acute abdominal pain. Emergency abdominal CT showed intussusception (Fig. 7). Gastrointestinal radiography revealed small bowel obstruction. The point of obstruction was located at the left lower abdomen (Fig. 8). Because there was no intussusception found on abdominal CT before the operation, and intussusception was not found during the operation, we think that the removal of the LT caused the intussusception. Another surgical exploration was carried out. During the exploration, severe small bowel dilatation located at 1.5 m from the ligament of Treitz was found, and part of the small intestines was found slide inside the nearby part. But the color of the intestine wall did not change. Thus, no necrosis was diagnosed (Fig. 9). A manual reduction was performed. After successful reduction, the intussusception length of about 70 cm was discovered. The patient was discharged on the 6th day after the operation.

## Discussion and conclusions

Intussusception mainly occurs in infants and children. Typical manifestations of intussusception involve abdominal pain, bloody stool, and abdominal mass [2]. Adult intussusception is rare, which only accounts for 5 % of all intussusception cases. The clinical manifestations of adult intussusception are not as typical as in children. Signs of adult intussusception were recurrent abdominal pain only. Bloody stool and abdominal mass were rarely reported [3]. About 90 % of intussusception in children is primary, while more than 90 % of intussusception in adults is secondary [4]. The tumor is the main cause of intussusception in adults, accounting for 63–77 % of the total cases. Malignant tumors are the most common cause of intussusception caused by tumors (50–73 %) [5]. The mechanism is mainly due to intestinal strictures induced by intestinal tumor, which results in incomplete intestinal obstruction. The peristaltic wave was interrupted and blocked at the site of the tumor, and local irritation occurred frequently. Disruption of the peristaltic rhythm, increased force of peristaltic contraction, movement of the tumor with peristalsis, or forward movement of intestinal contents may all result in intussusception [6]. Postoperative-related factors are the second major cause of intussusception in adults, such as postoperative abdominal adhesion and intestinal ostomy. Intussusception caused by the removal of the LT is rare and has never been reported.

Early diagnosis of intussusception in adults is difficult, and it might be misdiagnosed in an emergency easily. The patient in this case report presented with abdominal pain, reduced flatus passage, and frequency of bowel movement. So, it is difficult to make a definite diagnosis based on the signs and symptoms. CT plays an important role in the diagnosis of intussusception in adults. Intussusception can be diagnosed according to specific signs detected on the image of a CT scan, such as “concentric circle sign”, “comet tail sign” or “kidney-shaped sign”. These signs represent anatomic relationships between the layers of the intestinal wall, the intestinal lumens, and the mesentery. In this case, a “concentric circle sign” in the CT imaging was found, which greatly confirmed the working diagnosis [7].

This case of intussusception is due to the removal of the LT, and there is no relevant literature published at present. We hypothesized that the main causes of the intussusception, in this case, are: (1) The speed of tube withdrawal was too fast. (2) The water in the balloon of the LT was not evacuated completely.

Therefore, we suggest that the following steps should be done before removing the tube: (1) During the process of removal, the speed of tube withdrawal should be slow. The tube could even be withdrawn by a section of length each day until it is completely removed. (2) Before removing the LT, the health care professional should make sure to empty the water balloon completely. The purpose of these instructions is to ensure the intestines have sufficient time to adapt to the removal process and to prevent intussusception.

Once the diagnosis of adult intussusception is confirmed, surgical treatment is recommended: (1) If there was no necrosis, a manual reduction should be carried out. After the reduction, the bowel should be carefully examined for tumor, polyp, diverticulum, focal necrosis, and other types of lesions. (2) If necrosis of the intestine is found, the manual reduction is not recommended. An intestinal resection should be performed promptly. (3) To avoid squeezing, which can cause the spread of cancer cells to the intestine or bloodstream, patients with suspected malignant tumors should not receive the manual reduction. Intestinal resection and lymph node dissection should be performed instead. After the lesion is removed, the small intestine can be directly anastomosed. In this case, the patient’s intestine was not necrotic. So, a manual reduction was performed [8].

Medical risks are ubiquitous. Health care professionals should be cautious with every step in the medical procedure. This way, the incidence of iatrogenic complications will be minimized. This largely prevents patients from unnecessary suffering.

## Data Availability

Not applicable.

## Abbreviations

LT: Long intestinal tube
CT: Computed tomography
N: Neutrophils
WBC: White blood cell
HGB: Hemoglobin
CRP: C-reactive protein
CEA: Carcinoembryonic antigen
CA: Carbohydrate antigen

## References

[1] Ondhia Meraj N, Al-Mutawa Yousef H, Srikrishna, Losty Paul DIntussusception (2020). A 14-year experience at a UK tertiary referral centre. J Pediatr Surg.

[2] Ferrantella Anthony Q, Kirby P, Joshua (2020). Incidence of recurrent intussusception in young children: A nationwide readmissions analysis. J Pediatr Surg.

[3] Woo PJ, Am SG, Hoon BD (2010). Adult ileocolic intussusception caused by diffuse large B cell lymphoma. Korean J Gastroenterol.

[4] Kao Y-K, Chen J-H. Adult Jejuno-jejunal intussusception due to inflammatory fibroid polyp: a case report and literature review. Medicine (Baltimore).2019; 99(36): e22080. doi:10.1097/MD.0000000000022080. 10.1097/MD.0000000000022080 PMC7478680 32899081

[5] Hong KD, Kim J, Ji W, Wexner SD (2019). Adult intussusception: a systematic review and meta-analysis. Tech Coloproctol.

[6] Al-Radaideh AM, Omari HZ, Bani-Hani KE (2018). Adult intussusception: a 14-year retrospective study of clinical assessment and computed tomography diagnosis. Acta Gastroenterol Belg.

[7] Lief K, Gnananandan J, Calum C, Coffey D (2019). Diagnostic challenge of the non-specific presentation of adult intussusception. BMJ Case Rep.

[8] de Clerck F, Vanderstraeten E, De Vos M, Van Steenkiste C (2016). Adult intussusception: 10-year experience in two Belgian centres. Acta Gastroenterol Belg.

