# Correction to: A case of iatrogenic intussusception in adults: a rare case report

**DOI:** 10.1186/s12893-021-01299-9

**Published:** 2021-06-30

**Authors:** Qiang Hu, Yuanshui Sun, Jianfeng Shi

**Affiliations:** Department of General Surgery, Tongde Hospital of Zhejiang Province, 234 Gucui RD, Hangzhou, 310012 China

## Correction to: BMC Surg (2021) 21:271 https://doi.org/10.1186/s12893-021-01268-2

Following the publication of the original article [1], we have been notified that Jianfeng Shi was incorrectly listed twice in the authorship list, once in the second position and incorrectly spelled as Jinfeng Shi, and once in the last position. The incorrect second position has been removed.

The original article has been corrected.
